# Vitamin D Modulation of TRAIL Expression in Human Milk and Mammary Epithelial Cells

**DOI:** 10.1038/s41598-017-04521-y

**Published:** 2017-06-28

**Authors:** Yuvaraj Sambandam, Sakamuri V. Reddy, Jennifer L. Mulligan, Christina Voelkel-Johnson, Carol L. Wagner

**Affiliations:** 1 Department of Pediatrics, Darby Children’s Research Institute, Medical University of South Carolina, Charleston, SC 29425 USA; 2 Department of Otolaryngology, Medical University of South Carolina, Charleston, SC 29425 USA; 3 Department of Microbiology & Immunology, Medical University of South Carolina, Charleston, SC 29425 USA

## Abstract

The vitamin D levels in mothers affect the health status of both the mother and breastfeeding infant. Vitamin D deficient mothers’ infants are prone to rickets. While tumor necrosis factor-related apoptosis inducing ligand (TRAIL) has been implicated in cellular growth/apoptosis, immune cell function and bone-resorbing osteoclast formation, the expression of TRAIL in human milk as a function of vitamin D status in mothers remains unknown. We hypothesized that vitamin D deficiency alters TRAIL protein levels in human breast milk and mammary epithelial cells. Milk from vitamin D deficient mothers showed high levels of TRAIL (α and β) proteins compared to milk from vitamin D replete women. Western blot analysis of total cell lysate obtained from normal human mammary epithelial (HME-1) cells treated with variable doses (0–20 nM) of vitamin D for 24 h demonstrated that low levels (0.5 to 5 nM) significantly increased the TRAIL α but no change in β expression. In contrast, vitamin D at 20 nM concentration suppressed the expression of both TRAIL α and β proteins. Consistently, vitamin D regulated TRAIL mRNA expression in HME-1 cells. Our results indicate that vitamin D status in mothers modulates TRAIL expression in breast milk, which may have implications for both mother and infant health.

## Introduction

Vitamin D deficiency has been shown to play an important role in bone health and autoimmune diseases and adversely affects pregnancy and birth outcomes^1, 2^. It has been shown that vitamin D regulates calcium uptake, bone homeostasis, mammalian cell growth and division. In addition, vitamin D is implicated in the regulation of T-lymphocytes, monocytes and macrophages of the immune system^3–5^. Various factors such as less sunlight exposure due to indoor activity during the day or use of sunscreen, darker skin pigmentation, and health conditions such as pregnancy and obesity result in vitamin D deficiency in people^6^. Vitamin D deficiency during pregnancy has been shown to be associated with adverse health consequences such as osteomalacia, gestational diabetes, low phosphate levels and elevated levels of PTH in mothers as well as impaired bone development in children^7, 8^. The mammary epithelial cells' differentiation is critical to forming a functional mammary gland for infant breastfeeding﻿^9, 10^. It has been reported that vitamin D is involved in mammary gland development and lactation^11^. Studies have suggested that the vitamin D status of mother determines the vitamin D levels of breastfeeding infant^12–14^. Evidently, randomized controlled clinical trials indicated the beneficial role of maternal vitamin D supplementation on the vitamin D status of breastfed infants^14, 15^. Therefore, vitamin D deficient mothers are more likely to have infants with vitamin D deficiency which affects immune and bone health^16^. It has been reported that infants of vitamin D deficient mothers are prone to develop rickets^17^. However, it is unknown whether vitamin D deficiency in lactating mothers modulates the expression of cellular factor(s) in human milk that is associated with immune health and skeletal development in breastfeeding infants.

Tumor necrosis factor (TNF)-related apoptosis inducing ligand (TRAIL) is a member of the TNF superfamily^18^. TRAIL is detectable as a soluble protein in biological fluids^19^. TRAIL isoforms (α, β and ɣ) have been identified and implicated in cellular growth/differentiation^20^. Also, TRAIL induces apoptosis in malignant cells and at low doses could promote proliferation of normal cells^20^. TRAIL functions through death receptors DR4, DR5 which promote apoptosis and also binds to DcR1, DcR2 receptors that lack a death domain^21^. Similar to vitamin D, TRAIL has been shown to regulate immune cell functions, bone remodeling and cell growth/differentiation^20, 22^. However, the association between the levels of vitamin D in the maternal blood and TRAIL expression in human milk has not been previously described. Therefore, we hypothesize that vitamin D deficiency in mothers alters TRAIL expression in breast milk. In this study, we identified that milk samples collected from vitamin D deficient mothers contain elevated levels of TRAIL compared to the vitamin D replete group. Furthermore, our experiments revealed that vitamin D levels differentially regulates TRAIL isoform expression in mammary epithelial cells *in vitro*.

## Results

### Vitamin D deficiency in mothers increases TRAIL expression in breast milk

**Figure 1:**
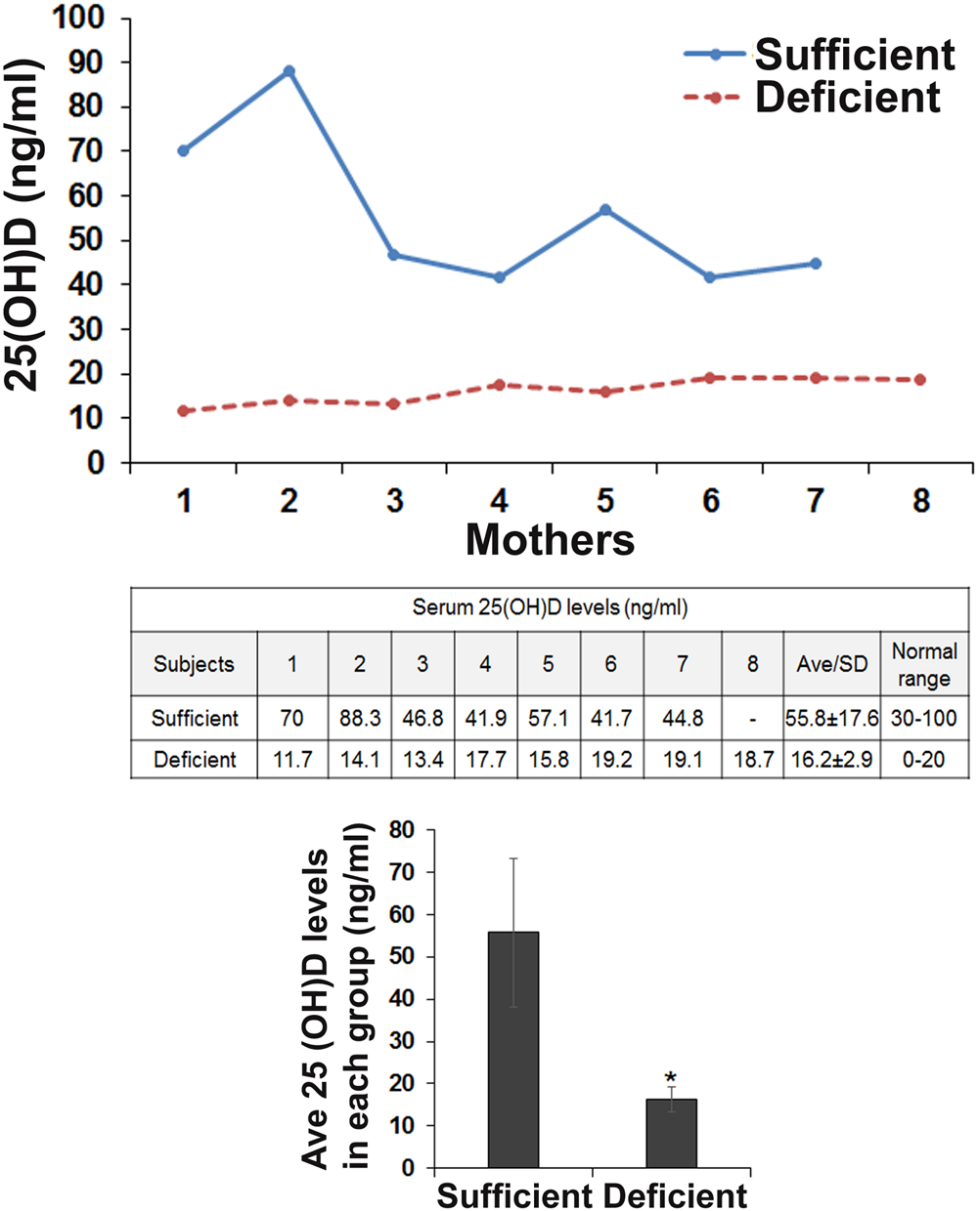
Serum 25(OH)D concentrations in mother groups. Serum samples collected from fifteen nursing mothers’ were measured for 25(OH)D levels to evaluate vitamin D status in mother groups as described in methods. Sufficiency: >30 ng/mL; Deficiency: <20 ng/mL. Each bar represents the mean ± SD (P < 0.05). *compared to control sufficient group.

**Figure 2:**
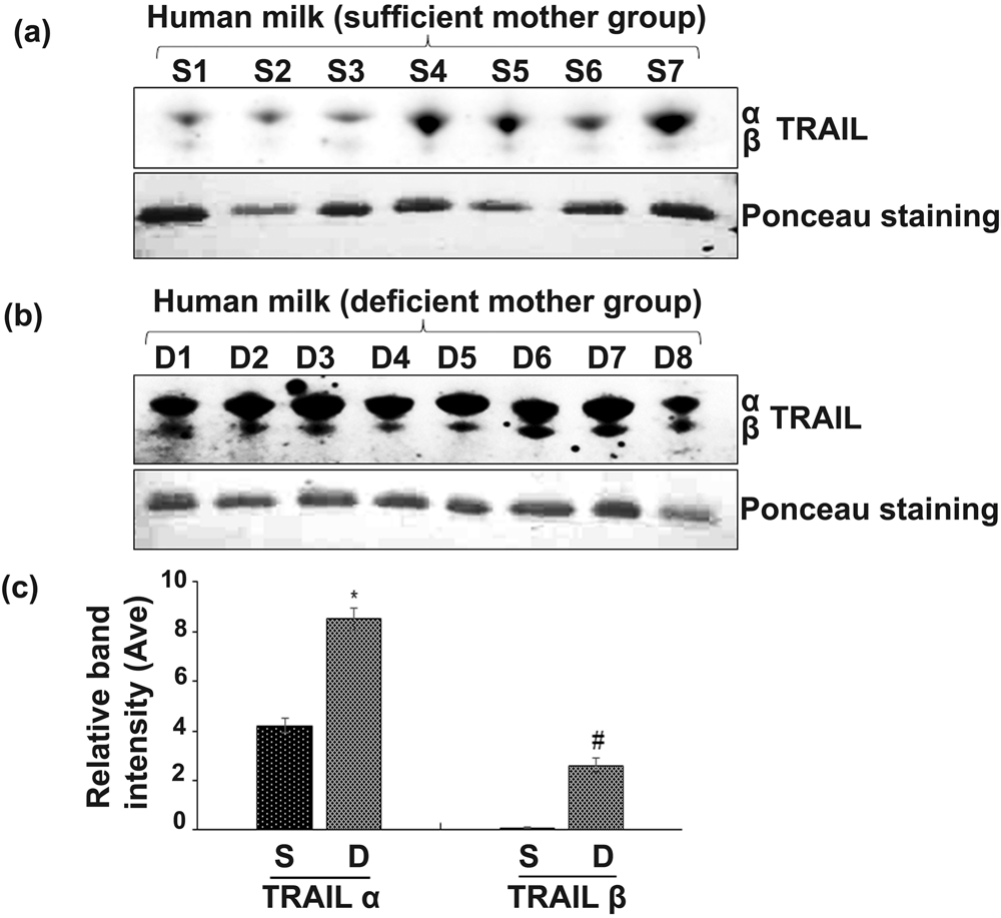
TRAIL expression in human breast milk. Milk samples from vitamin D sufficient and deficient mother groups containing an equal amount of protein (50 µg) were subjected to western blot analysis for TRAIL expression using a specific anti-TRAIL antibody. Ponceau staining of the membrane was performed as a loading control (**a** and **b**). The band intensity was quantified by NIH ImageJ. The relative band intensity was normalized by band detected by Ponceau staining (**c**). *TRAIL α compared to sufficient group; #TRAIL β compared to sufficient group. The values are expressed as mean ± SD (*p < 0.05). S- sufficient; D- deficient. *compared to 0 nM of 1,25(OH)_﻿2_D treatment. (full-length blots are included in the Supplementary Information Figs 1 and 2).

Physiologic levels of vitamin D has been implicated as an important stimulant for innate immunity and bone remodeling. TRAIL expression has been shown to be upregulated in monocytes and macrophages in response to immune cytokines such as interferon-β (IFN-β)^23, 24^. Vitamin D and TRAIL have been associated with modulation of immune function and bone health. However, their relationship to one another during lactation has not been described. Therefore, serum samples from fifteen breastfeeding mothers aged 20–38 years that included seven Caucasian, seven African-American, and one Hispanic mother were analyzed. Serum 25(OH)D levels were measured to evaluate the active form of vitamin D [1,25(OH)_2_D] status in maternal blood as classified by the Endocrine Society clinical practice guideline^25^. Measurement of total circulating 25(OH)D concentrations identified seven samples with normal range (>30 ng/ml) and therefore considered vitamin D-sufficient group. There were eight mother’s serum samples which demonstrated low levels of 25(OH)D (<20 ng/ml) and were considered as a vitamin D deficient group (Fig. 1). Milk samples from 15 of the mothers (8 deficient and 7 sufficient) were analyzed for TRAIL expression by western blot. As shown in Fig. 2a–c, we identified TRAIL α expression in all the milk samples obtained from vitamin D sufficient mother group. However, TRAIL β expression is at very low levels in all the samples analyzed. Interestingly, milk from vitamin D deficient mothers showed elevated levels of TRAIL proteins (α and β) compared to milk from mothers with sufficient vitamin D levels. However, TRAIL ɣ protein is not detectable in human breast milk and mammary epithelial cells (data not shown). These results suggest that vitamin D status in mothers modulate TRAIL expression levels in human breast milk.

### Effect of vitamin D on TRAIL expression in human breast epithelial cells

**Figure 3:**
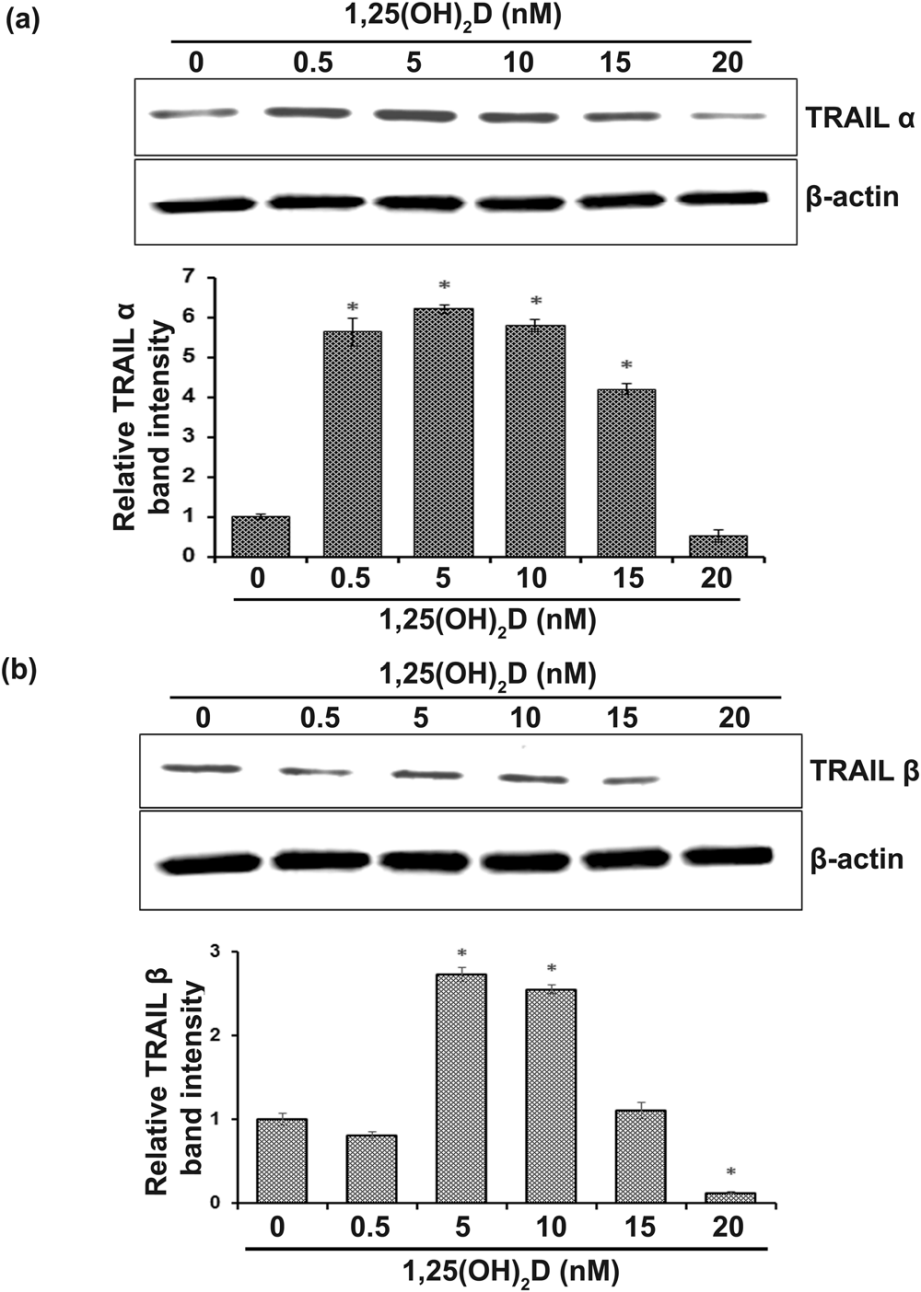
Western blot analysis for vitamin D [1,25(OH)_2_D] regulation of TRAIL isoforms expression in normal human breast epithelial cells. HME1 cells were treated with different concentrations of 1,25(OH)_2_D at 0–20 nM for 24 h. (**a** and **b**) Total cell lysates were subjected to western blot for TRAIL expression using a specific anti-TRAIL antibody. The band intensity was quantified by NIH ImageJ program. β-actin expression served as a control. The values are expressed as mean ± SD (*p < 0.05). (Full-length blot is included in the Supplementary Information Fig. 3).

**Figure 4:**
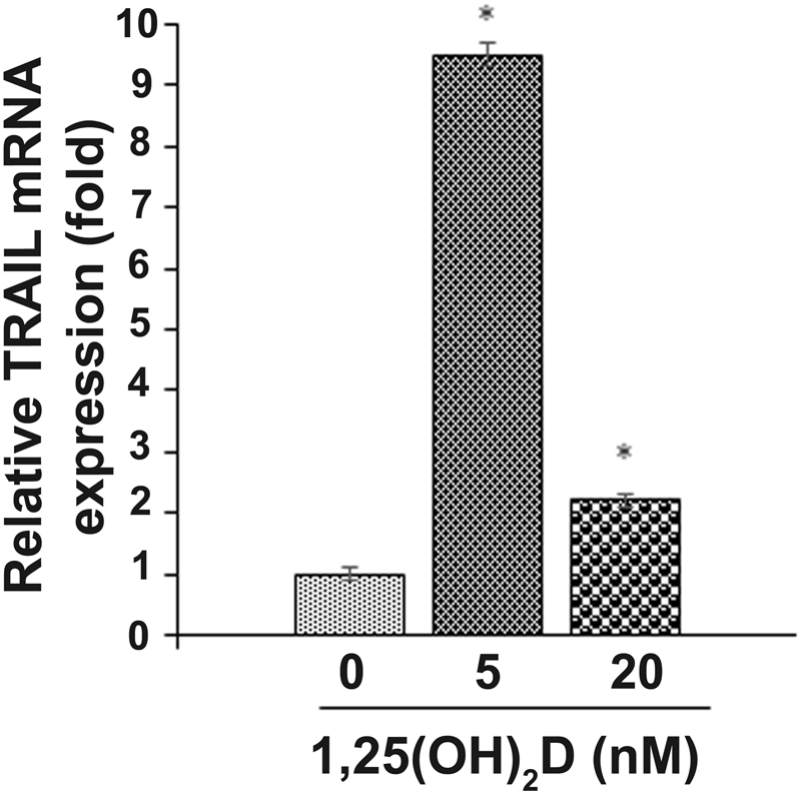
Vitamin D regulation of TRAIL mRNA expression in normal human breast epithelial cells. HME-1 cells were treated with vitamin D [1,25(OH)_2_D] at 0, 5 and 20 nM for 24 h. Total RNA isolated from these cells was subjected to real-time RT-PCR analysis for TRAIL mRNA expression as described. The relative mRNA expression was normalized on GAPDH amplification. The values are expressed as mean ± SD (*p < 0.05). *compared to 0 nM of 1,25(OH)_2_D treatment.

Since the expression of TRAIL is elevated in vitamin D deficient mothers’ breast milk, we next examined the direct effect of vitamin D on normal human breast epithelial cells to analyze the TRAIL expression. Although 25(OH)D is the main circulating metabolite, 1,25(OH)_2_D is the active form which responsible for most of the biological actions^26^. Therefore, HME-1 cells were treated with 1,25(OH)_2_D at variable concentrations (0–20 nM) as used previously^27^ for 24 h. Western blot analysis of total cell lysate obtained from these cells treated with low doses (0–5 nM) demonstrated increased TRAIL α (6-fold) expression. However, the levels of β expression did not alter. In contrast, 1,25(OH)_2_D at 10–20 nM concentrations dose-dependently suppressed both the TRAIL α and β protein expression (Fig. 3a,b). We further tested the TRAIL mRNA expression in breast epithelial cells following treatment with 1,25(OH)_2_D at selected doses (0, 0.5 and 20 nM) for 24 h. Real-time RT-PCR analysis of total RNA isolated from the cells showed a 9-fold increase at 5 nM concentration of 1,25(OH)_2_D compared to untreated control cells. Conversely, 1,25(OH)_2_D at high dose (20 nM) inhibited TRAIL mRNA expression (Fig. 4). These results indicate that the active form of vitamin D regulates TRAIL expression in normal human breast epithelial cells.

## Discussion

This study demonstrated that breast milk from vitamin D deficient mothers contains high levels of TRAIL (α and β) proteins compared to sufficient mother group. Further, we identified that low doses of the active form of vitamin D (0.5 to 5 nM) treatment to HME-1 cells increased the TRAIL α but no change in β expression. However, 1,25(OH)_2_D at high doses (20 nM) suppressed the expression of both TRAIL α and β proteins. Therefore, our results indicate that TRAIL is expressed in breast milk as a function of vitamin D status in mothers. Vitamin D is involved in immune cell function and activation^28^. Vitamin D supplementation of mothers results in vitamin D sufficiency in breast-fed infants^13^, thus potentially modulating the immune signature of maternal breast milk to her recipient infant. It has been reported that human soluble TRAIL is an immune regulator present in human milk and supports the infant immune system^29^. Thus, our findings that vitamin D is associated with altered TRAIL expression in breast milk may be an important link to the mother and infant immune system. Evidence suggests that vitamin D deficiency could be carcinogenic to the brains of offspring in mouse model^30^. TRAIL present in human milk has been shown to play a role in anti-cancer activity^31^. Since there is no TRAIL toxicity demonstrated *in vitro* and *in vivo* treatment^32, 33^, it is possible that TRAIL expression in the milk of mothers with vitamin D deficiency could be a compensatory mechanism to protect infant health. The functional role of vitamin D in fetus and infant’s bone development has been previously demonstrated. Vitamin D influences the acquisition of bone mineralization in the rapidly growing fetal skeleton^34^. Mother-offspring group studies have shown that maternal vitamin D insufficiency leads to reduced bone mass during childhood^35, 36^. We recently demonstrated that TRAIL enhances bone-resorbing osteoclast differentiation^37, 38^. Therefore, elevated levels of TRAIL expression due to vitamin D deficiency may have a significant impact on both mother and infant bone growth/development.

Vitamin D at sufficient levels has been implicated in the protection of the immune system^28^. TRAIL has been shown to be upregulated by lipopolysaccharide (LPS) and IFN-β produced by immune cells^39^. Therefore, it is possible that mammary cells, as well as immune cells, can contribute to TRAIL expression in human breast milk. Previously, it has been shown that vitamin D deficiency during pregnancy increases genotoxic risks in newborns^40^. Vitamin D deficiency during pregnancy may also be associated with smaller fetal thymus and systemic inflammatory response^41^. Similarly, it has been shown that early exposure to TRAIL causes a loss of thymic cellularity and neutralizes all acute inflammatory responses in mice^42, 43^. This evidence suggests that vitamin D and TRAIL expression in mammalian cells play a major role in both mother and infant immune health. Therefore, specific modulators of the TRAIL signaling axis and vitamin D analogs may lead to the development of randomized clinical trials to rescue from the consequences of vitamin D deficiency in both mother and infant. Also, these studies may have implications regarding vitamin D supplemention during lactation. However, this study should be performed in lactating women with diverse ethnic groups in a large population.

TRAIL has been shown to increase normal mammalian cell growth and induce apoptosis in cancer cells. In contrast, 25(OH)D inhibits the growth of the human mammary epithelial cells^44^. Therefore, it is possible that elevated levels of TRAIL influence vitamin D metabolism in mammary epithelial cells^45^. High dose vitamin D decreased TRAIL expression compared to basal levels, suggesting that low levels of vitamin D may be required to stimulate TRAIL expression in mammary epithelial cells. It is likely that the down-regulation of the TRAIL-α and β in response to high doses (>20 nM) of vitamin D could be a compensatory mechanism to protect the mammary cells. Thus, our results suggest that the vitamin D modulation of TRAIL expression in mammary epithelial cells may contribute to TRAIL protein levels in human milk, which may have implications for both mother and infant health under vitamin D deficient and sufficient conditions.

## Methods

### Serum and breast milk sample collection

All the experimental protocols were approved by IRB (HR#10727) at the Medical University of South Carolina and all methods were performed in accordance with the relevant guidelines and regulations. The collection of breast milk and serum samples from women participating in an NICHD-sponsored study of vitamin D supplementation during lactation was carried out by the approved guidelines. All subjects were taking a prenatal vitamin with 400 IU of vitamin D. Blood samples were collected by venipuncture at the time of the study visit; then placed in a serum separator tube, centrifuged, and serum stored at −20 °C until later analysis. Milk samples were collected using an electric breast pump. Human milk was collected from the left breast directly into a small sampling bottle at the regular feeding time in the morning, 2 h after the previous breastfeed for consistency of results. The samples were stored in a −20 °C freezer for subsequent analysis.

### Quantification of circulating 25(OH)D

Concentrations of circulating 25(OH)D were quantified by chemiluminescent immunoassay (DiaSorin Co., Ltd., Stillwater, MN) as described^46, 47^. Total circulating serum 25(OH)D concentration was measured in duplicate within the month the blood sample was obtained. Vitamin D sufficiency was defined as a total circulating 25(OH)D concentration of >30 ng/mL and deficiency as <20 ng/mL as per the Endocrine Society clinical practice guidelines^25^.

### Normal human breast epithelial cell culture

Normal human breast epithelial (HME-1) cells were maintained in Clonetics® MEBM® Mammary epithelial basal medium (MEBM) supplemented with bovine pituitary extract, GA-1000, human epidermal growth factor, insulin, and hydrocortisone (Lonza, Walkersville, MD). Cells were cultured in a humidified chamber with 5% CO_2_ and 95% air at 37 °C.

### Western blot analysis

Total protein content of the milk samples from vitamin D sufficient and deficient women and HME-1 cells stimulated with 0–20 nM of vitamin D [1,25(OH)_2_D]^48, 49^ was measured using the Bradford protein assay method (Bio-Rad, Hercules, CA). Protein (50 μg) samples were then subjected to SDS-PAGE using 4–12% Tris–HCl gels and blot transferred onto a nitrocellulose membrane. Blocking was performed with 3% bovine serum albumin in TBS-T (50 mM Tris, pH 7.2, 150 mM NaCl; 0.1% Tween 20) for 1 h followed by incubation with anti-TRAIL (Abcam Inc., Cambridge, MA) overnight at 4 °C. The blots were then incubated for 1 h with HRP-conjugated secondary antibody and developed using ECL system. The analysis of β-actin expression levels and Ponceau staining of the membrane was performed as a loading control. The band intensities were quantified by NIH ImageJ program.

### Real-time RT-PCR

Total RNA was isolated from HME-1 cells treated with different concentrations (0, 5 and 20 nM) of 1,25(OH)_2_D for 24 h using RNAzol reagent (Molecular Research Center, Inc. Cincinnati, OH). The reverse transcription reaction was performed using poly-dT primer and moloney murine leukemia virus reverse transcriptase (Applied Biosystems, Foster City, CA) in a 25 μl reaction volume containing total RNA (2 μg), 1× PCR buffer and 2 mM MgCl_2_, at 42 °C for 15 min followed by 95 °C for 5 min. The quantitative real-time RT-PCR was performed using IQ^™^ SYBR Green Supermix in an iCycler (iCycler iQ Single-color real-time-PCR detection system; Bio-Rad, Hercules, CA). The primer sequences used to amplify human glyceraldehyde-3-phosphate dehydrogenase (hGAPDH) mRNA were sense 5′-CCT ACC CCC AAT GTA TCC GTT GTG-3 and anti-sense 5′-GGA GGA ATG GGA GTT GCT GTT GAA-3′; TRAIL mRNA primer sequences were sense 5′-TTC ACA GTG CTC CTG CAG TC-3′ and antisense 5′-CAG CAG GGG CTG TTC ATA CT-3′. Thermal cycling parameters were 94 °C for 4 min, followed by 35 cycles of amplifications at 94 °C for 30 s, 58 °C for 1 min, 72 °C for 2 min, and 72 °C for 10 min as the final elongation step. Relative levels of mRNA expression were normalized in all the samples analyzed on the levels of GAPDH amplification.

### Statistical analysis

Results are represented as mean ± SD of at least three independent experiments. Differences between experimental groups were analyzed by one-way ANOVA and Tukey test using GraphPad Prism software (GraphPad Software, Inc, La Jolla, CA). Values were considered significantly different at p < 0.05.

## Electronic supplementary material

Supplementary Info
