# Supplementary material for: Vitamin D Modulation of TRAIL Expression in Human Milk and Mammary Epithelial Cells
Source: Sci Rep. 2017 Jun 28;7:4362. doi: 10.1038/s41598-017-04521-y (PMC5489519; doi:10.1038/s41598-017-04521-y)
Supplement: Supplementary file 1 — Supplementary Info [file 41598_2017_4521_MOESM1_ESM.pdf]

Vitamin D Modulation of TRAIL Expression in Human Milk and Mammary Epithelial Cells

Yuvaraj Sambandam[1], Sakamuri V. Reddy[1], Jennifer L. Mulligan[2], Christina Voelkel-Johnson[3] and Carol L. Wagner[1*]

[1]Department of Pediatrics, Darby Children's Research Institute; [2]Department of Otolaryngology; [3]Dept. of Microbiology & Immunology, Medical University of South Carolina, Charleston, SC 29425, USA.

*Address for correspondence: Carol L. Wagner, MD, Darby Children’s Research Institute, 173 Ashley Avenue, Charleston, SC 29425. Tel. Ph: 843-792-8829; Fax: 843-792-8801; E-mail: wagnercl@musc.edu

Supplementary figures

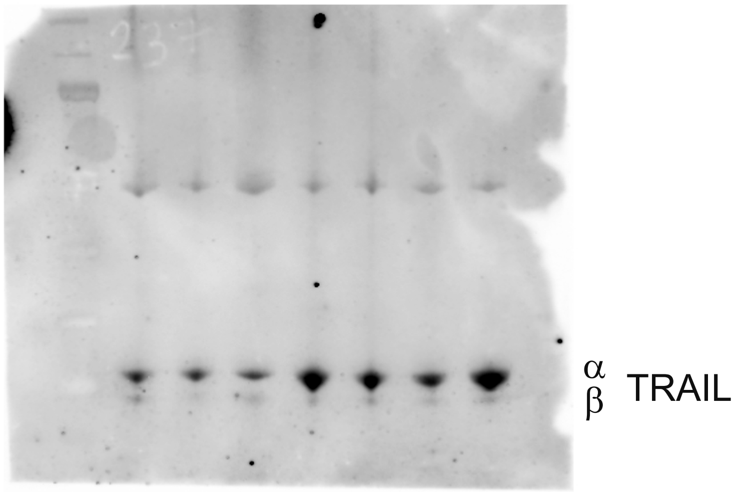

Fig.1. Western blot of milk samples from vitamin D sufficient mothers

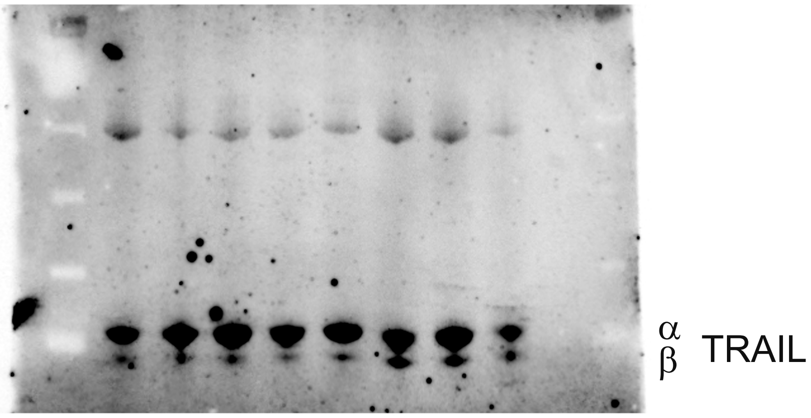

Fig.2. Western blot of milk samples from vitamin D deficient mothers

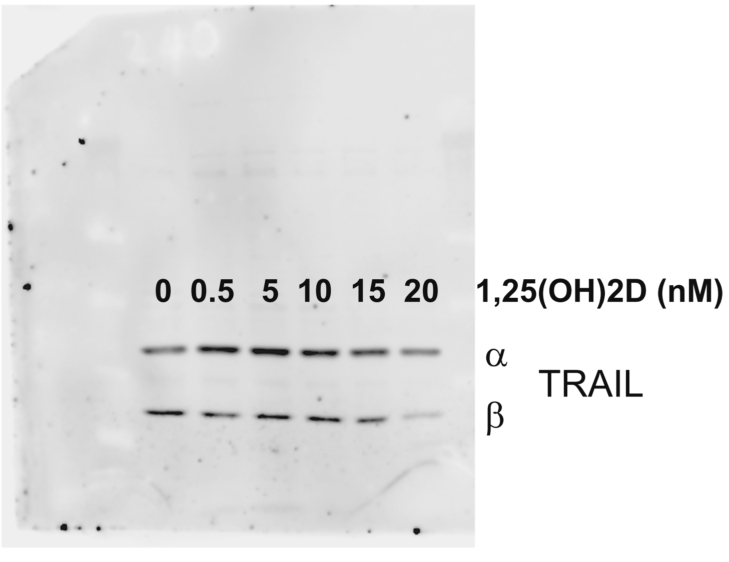

Fig.3. Western blot of normal breast epithelial cells treated with vitamin D
